# Infirmary Patients Sleeping on the Floor

**Published:** 1912-01-27

**Authors:** John R. Staddon

**Affiliations:** Medical Officer, Ipswich Workhouse. 6 Silent Street, Ipswich


### The Editor's Letter Box.

### INFIRMARY PATIENTS SLEEPING ON THE FLOOR.

Sir,—*Re* your report as to the congested condition of Ipswich Workhouse Infirmary. It is a pity that you did not make some inquiry before you wrote the paragraph in The Hospital. Had you done so, you would have found that for the last six years I have repeatedly called the Guardians' attention to the necessity of providing more accommodation for the sick and infirm, and have been backed up by the Local Government Inspector. Like a good many others boards, they think that the present legislation is likely to diminish pauperism, but will find out their mistake.

I hope you will correct the errors in your paragraph.—Yours truly, John R. Staddon,
Medical Officer, Ipswich Workhouse.

6 Silent Street, Ipswich, January 17, 1912.

[We are very glad our paragraph last week has given Mr. Staddon the opportunity of writing and ourselves of publishing this letter. Its significance we deal with in our Notes this week.—Ed. The Hospital.]

